# Indoor air quality and its impacts on asthma and COPD

**DOI:** 10.1136/bmjresp-2025-003807

**Published:** 2026-03-18

**Authors:** Tun Zan Maung, Rose Aning, Michael Newnham, Eleanor Holt, Christian Pfrang, Alice Margaret Turner

**Affiliations:** 1 University of Birmingham, Birmingham, UK; 2 Department of Respiratory Therapy, University of Ghana, Accra, Ghana; 3 School of Heath Sciences, University of Birmingham, Birmingham, UK; 4 School of Geography, Earth and Environmental Sciences, University of Birmingham, Birmingham, UK

**Keywords:** Asthma, Pulmonary Disease, Chronic Obstructive, Emphysema

## Abstract

**Background:**

Though indoor air pollution is associated with high mortality and economic impact globally, it is relatively understudied. Knowledge gaps remain regarding exposure to peak pollutant concentrations and their effects, especially among patients with respiratory diseases who are susceptible to a greater impact.

**Methods:**

This 2-week cohort study monitored indoor air quality and symptoms in patients with asthma and chronic obstructive pulmonary disease. Statistical process control charts were used to track hourly pollutant peaks, while notched box plots visualised significant particulate matter 2.5 (PM_2.5_) peaks over 6-hour periods. Linear mixed-effects and autoregressive models were used to assess the impact of PM_2.5_ on symptoms.

**Results:**

The analyses included 30 participants. Hourly plots revealed that 43.3% experienced PM_2.5_ and PM_1_ peaks above the upper control limit between 6 pm and 9 pm, with 33.3% occurring specifically at 19:00 hours, consistent with cooking as a source of particulates. There were also a few peaks between 10 am and 12 noon. Peaks recorded between midnight and 5 am were minimal, corresponding to low activity during sleep. Smokers exhibited higher average pollutant levels than non-smokers. On average, participants experienced four to six pollutant peak periods exceeding the WHO 2021 air quality guidelines. No statistically significant association was found between PM_2.5_ and asthma symptoms (p>0.05), although a weak relationship was observed visually.

**Conclusion:**

The data suggest that human activities significantly influence indoor air quality for PM, indicating that behavioural interventions could help optimise it.

**WHAT IS ALREADY KNOWN ON THIS TOPIC**

- Indoor air pollution exposure poses a significant health threat because people spend most of their time indoors, yet it remains relatively understudied, particularly among the most vulnerable to its effects—patients with respiratory disease.

**WHAT THIS STUDY ADDS**

- This study revealed that fluctuating patterns of high concentrations of multiple pollutants simultaneously observed were largely driven by human activities, particularly cooking during the evening. It was also found that the patients with chronic obstructive pulmonary disease and asthma in the study were generally exposed to pollutant levels exceeding the WHO air quality guidelines for both short-term and long-term exposures, but symptom variations did not consistently relate to pollutant exposure patterns.

**HOW THIS STUDY MIGHT AFFECT RESEARCH, PRACTICE OR POLICY**

- Based on the finding that human activities likely contributed to indoor air quality, focusing on behavioural interventions, perhaps centred on cooking practices, may optimise this and further research in larger cohorts to confirm the findings, and with patients to codesign appropriate behaviour change interventions is needed.

## Introduction

 The detrimental effects of air pollution on human health are substantial and globally contribute to 7 million premature deaths per year, 91.5 million disability-adjusted life years specifically for household air pollution and an economic impact of $8.1 trillion.^1^,^3^ Harmful pollutants such as nitrogen dioxide (NO_2_), volatile organic compounds (VOCs) and particulate matter (PM) have become a significant threat to our environment and well-being.^4^ Initiatives to combat outdoor air pollution have been underway, and recent analyses of pollution emission trends, particularly in the UK, demonstrate policy initiatives have resulted in the decline of the concentrations of several studied pollutants within about 50 years.^5^ Although outdoor air quality has been improving, indoor air pollution has emerged as an area of rising concern due to more airtight buildings that may trap indoor emissions due to reduced air exchange with outdoors, indoor activities such as cooking and heating and a greater amount of time spent indoors.^6^,^8^ However, there are gaps in our understanding of concentrations of pollutants indoors. Although individuals in the UK spend up to 90% of their time indoors (primarily within their own homes), research and interventions have predominantly focused on outdoor air pollution.^7^ Understanding the sources and impacts of indoor environments could be crucial for developing practical solutions to address indoor air quality challenges.

In 2020, 3.2 million deaths worldwide were attributed to household air pollution, with approximately 40% of these deaths linked to respiratory conditions.^9^ Per the 2021 Global Burden of Disease study, nearly 34% of the world’s population was exposed to household air pollution.^10^ Individuals with pre-existing respiratory conditions (eg, asthma and chronic obstructive pulmonary disease (COPD)) are particularly vulnerable to adverse effects of poor indoor air quality.^9^ For example, short-term exposure to indoor PM_2.5_ increases the risk of hospitalisation for patients with COPD.^11^ This heightened susceptibility arises from the underlying pathophysiology of their conditions and from symptom-related limitations (such as dyspnoea), which may restrict outdoor activity and result in prolonged time spent indoors, thereby increasing their cumulative exposure to indoor air pollutants.^12^

Daily activities within homes, features of households and infiltration of outdoor air pollution sources contribute to indoor air pollutant levels.^8^ PM sources include activities such as cooking and smoking.^12 13^ High exposure to PM has been associated with clinically significant outcomes, including increased fractional exhaled nitric oxide levels, reduced peak expiratory flow rate (PEFR) and reduced forced expiratory volume in 1 s among individuals with asthma and COPD.^12^ Other pollutants, such as NO_2_, emitted from gas stoves and water heaters, have been found to worsen symptoms, including cough, exacerbation rates and hospitalisations.^13^ Meanwhile, household features such as floor levels, building materials and airtight construction contribute to the level of exposure to pollutants such as VOCs, emitted from household products, personal products and building materials.^8 12^

Our systematic review of indoor air quality and its effects on health identified a significant gap in the evidence about the impact of indoor air quality on respiratory patients.^12^ We hypothesised that higher levels of indoor air pollutants would be associated with greater symptom burden, particularly in individuals with asthma. The aim of this observational study was to investigate indoor pollutant exposure in patients with asthma or COPD, assess pollutant exposure trends and examine their relationship to symptoms and disease control.

## Methods and materials

### Study design and ethics

A short-term cohort study monitoring symptom burden and indoor air quality was conducted. Study participants were recruited from the Midlands region of the UK between November 2022 and October 2023 and were visited twice for data collection. Baseline data included demographics, respiratory diagnosis, clinician-defined disease severity (as reported by the participant’s physician and in line with national guidance),^14 15^ total number of respiratory exacerbations and hospitalisations due to respiratory disease in the past year, smoking history, occupational history and current medications. During the baseline visit, the research team set up the indoor air quality monitor and issued an e-diary or paper diary (if unable to use the e-version) for recording daily symptoms. After 14 days, a follow-up visit was completed, at which the air quality monitor and e-diary or paper diary were collected. A short-term study was chosen primarily so that feasibility of monitoring could be tested while generating usable data for planning of subsequent larger studies.

### Symptoms and disease control

Validated questionnaires, acceptable to and usable by patients, were used to collect daily symptoms over a 2-week period, using the Bronkotest score for patients with COPD and the Asthma Control Test (ACT) for patients with asthma (online supplemental tables S3 and S4). Patients with asthma were also given a peak expiratory flow metre to measure their daily PEFR.

### Indoor air quality

Levels of PM_10_, PM_2.5_, PM_1_, NO_2_ and VOCs in indoor air were measured by the Plume Labs Flow 2 monitor.^16^ Recordings were made continuously and linked to an internet-enabled mobile phone to transmit data to the research team, effectively providing minute-by-minute data. The performance of the air quality monitor was validated by colocation with a reference instrument at the Birmingham Air Quality Supersite (BAQS). However, data for NO_2_ and VOCs from the Flow 2 monitor differed substantially from BAQS data and were thus deemed unreliable. Hence, subsequent results focused on PM only (PM_10_, PM_2.5_, PM_1_), with the primary focus on PM_2.5_ since it penetrates deeper into the lung, systemically absorbed and linked to multiple features of airway disease.^17^ The device was installed in the location within the home where participants spent most of their time, and participants were further advised to record any activities they took that might alter indoor air quality, such as opening windows or using an extractor fan. In the case of equipment failure or gaps in recording, participants were contacted to repeat the study.

### Data analysis and statistics

The characteristics of the study cohort were described, being summarised as n (%) and mean (SD). Air quality was explored within participants by visualising longitudinal data day by day to see whether there were consistent trends between participants. Similar plots using hourly time periods were plotted as statistical process control charts to see timing and number of peaks occurring beyond day-to-day norms of that individual (online supplemental figures S2 and S3). The average values of the upper control limits (UCL), defined as 3 SDs from the mean, of the various pollutants for all the participants and the average number of peaks counted for all the pollutants for all the participants were compared with the current WHO annual average and 24-hour average air quality guidelines to appreciate the clinical importance of transient peaks and the implications of frequent peaks above such values (online supplemental table S5).^18^ Further visualisation of frequency of significant PM_2.5_ peaks during portions of the day was achieved by use of notched box plots with 95% CI (due to skewed data) for periods of 6 hours (24:00 to 06:00, 06:00–12:00, 12:00–18:00, 18:00–24:00). The association between PM_2.5_ and lagged (by 1 day) symptoms/disease control was investigated visually, and with mixed linear models where time and individual were random effects; an autoregressive correlation structure was included to account for the likelihood that values from the same individual were likely to be correlated. Unlagged models were completed for comparison (online supplemental table S1). Adjustment for additional confounders was not possible due to low numbers.

### Patient and public involvement

The University of Birmingham Respiratory Patient Advisory Group was consulted about the overall aims of the project, the structure of the patient information sheets and to aid recruitment to the study. The wider grant engaged the public and other stakeholders through a series of events that inquired about indoor air quality.

## Results

### Patient characteristics

38 patients consented to the study, but eight patients had large amounts of incomplete symptom or air quality data, despite attempts to repeat measures. Only the 30 participants with complete data were included in the reported analyses (n=20 asthma, n=10 COPD); their characteristics are presented in table 1. Individuals with asthma were younger than patients with COPD (mean age 56 vs 67), and there were more ‘never smokers’ among the asthma participants (55% vs 0%). Most participants were at the severe stage of their disease (70% of the asthma participants were at the severe stage and 70% of the COPD participants as well).

**Table 1:** Summary of the demographic characteristics of patients with asthma and COPD in the study

Demographic characteristic of participants	Categories	Asthma (n=20)	COPD (n=10)
Age*		56 (±13.7)	67 (±9.4)
Sex	Male	5 (25)	5 (50)
	Female	15 (75)	5 (50)
Ethnicity	White/British or Irish	18 (90)	9 (90)
	Asian/Asian British	1 (5)	0 (0)
	Black/Caribbean	1 (5)	0 (0)
	Other	0 (0)	1 (10)
Disease severity	Mild	2 (10)	1 (10)
	Moderate	4 (20)	2 (20)
	Severe	14 (70)	7 (70)
Medications	SMART	11 (55)	2 (20)
	SABA	3 (15)	2 (20)
	Corticosteroid	2 (10)	1 (10)
	LABA/ICS combination	4 (20)	0 (0)
	LAMA	6 (30)	0 (0)
	LAMA/LABA combination	0 (0)	1 (10)
	LAMA/LABA/ICS combination	0 (0)	1 (10)
	LTRA	8 (40)	0 (0)
	Theophylline	1 (5)	0 (0)
	Biologic	7 (35)	0 (0)
	Mucolytic	8 (40)	2 (20)
	None	1 (5)	4 (40)
Employment	Active	7 (35)	3 (30)
	Retired	7 (35)	5 (50)
	Unemployed	6 (30)	2 (20)
Smoking history	Current smoker	0 (0)	3 (30)
	Ex-smoker	9 (45)	7 (70)
	Never smoked	11 (55)	0 (0)
Comorbidities	None	7 (35)	2 (20)
	Multimorbidities	14 (65)	8 (80)
Baseline symptom scores	ACT (asthma)	15 (±5.1)	–
	Bronkotest (COPD)	–	19 (±7.6)
Exacerbations in the last year	0	5 (25)	1 (10)
	1	3 (15)	1 (10)
	2	4 (20)	4 (40)
	≥3	8 (40)	4 (40)

* The findings are presented as n (%) for all the categories except for the age and the baseline symptom scores (mean (±SD)).

ACT, Asthma Control Test; COPD, chronic obstructive pulmonary disease; ICS, inhaled corticosteroids; LABA, long-acting beta agonist; LAMA, long-acting muscarinic antagonist; LTRA, leukotriene receptor antagonist; SABA, short-acting beta agonist; SMART, single maintenance and reliever therapy.

### Trends and patterns of indoor air pollutants

Hourly plots (representative examples in online supplemental figures S2 and S3) revealed some periods when peaks above the UCL were recorded. Between 6 pm and 9 pm, 43.3% of participants had PM_2.5_ and PM_1_ above the UCL, and 33.3% were specifically seen at 7 pm (table 2), consistent with cooking as a source of particulates; patterns of data for PM_2.5_ and PM_1_ were very similar (online supplemental figure S4), but PM_10_ showed less clear diurnal variability (for counts and times of peaks above the UCL for all participants, see online supplemental table S2). The number of pollutant peaks recorded between 00:00 and 05:00 was very low, consistent with low human activity during sleep. This was the case for most participants and all the pollutants studied (online supplemental figure S4).

**Table 2:** A summary of the notable periods within the day when peaks of PM_2.5_ and PM_1_ were observed among participants

Hour/time interval	Participants, n (%)
7 am–10 am	12 (40)
12 pm–5 pm	14 (46.7)
6 pm–9 pm	13 (43.3)
7 pm	10 (33.3)

PM, particulate matter.

Smokers had high mean values on their hourly plots (20 µg/m^3^—nearly 40 µg/m^3^), being greater than the upper limit values of some of the non-smokers (online supplemental figures S2 and S3 and table S2), with further peaks usually 40–60 µg/m^3^ and a maximum of 123.15 µg/m^3^. These relatively high upper limit values were not significant events for these patients, but rather ‘normal’ according to their specific individual daily routines. Therefore, they were not flagged by upper limit peak analyses (online supplemental figure S3).

### Clinical importance of pollutant levels among participants

For the pollutants with current thresholds of exposure set by WHO (PM_10_ and PM_2.5_), mean values at peak exposure (as defined by upper limits (UCLs)) for all participants exceeded WHO air quality guidelines.^18^ Based on the number of peaks above the UCLs, on an average of four to about six occasions within the study period, participants were exposed to pollutant levels above WHO 2021 air quality guidelines (online supplemental table S5).

### Relationship between pollutants and symptom scores

**Figure 1:**
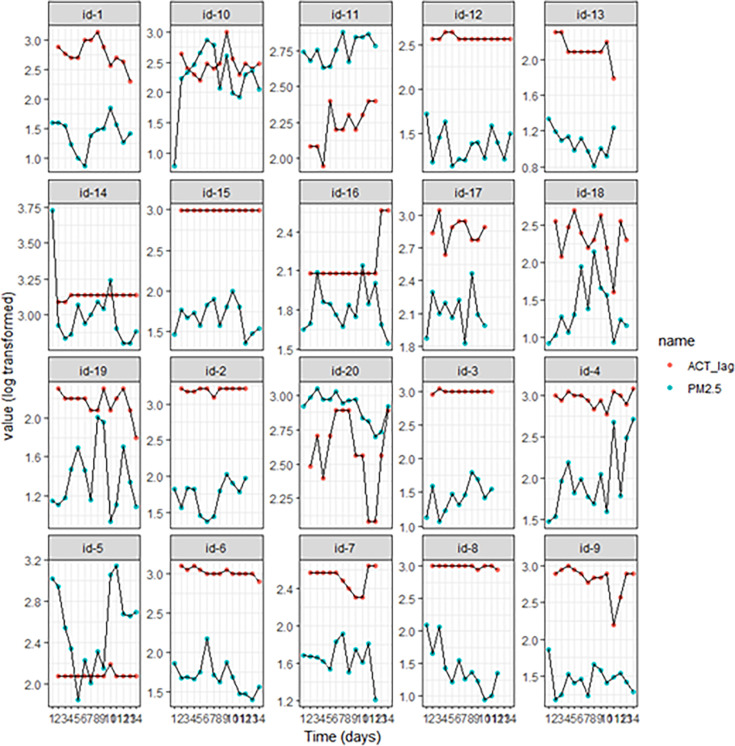
Time course monitoring of particulate matter (PM_2.5_) plotted against daily Asthma Control Test (ACT) scores of participants. Each plot represents the daily average of PM_2.5_ measurements recorded and the daily record of ACT scores for each participant for the study period. The values of PM_2.5_ and ACT were log transformed to aid visualisation of one plot and to identify the time trends. (Refer to the online supplemental results for comparable figures and tables for actual values of PM_2.5_.)

The time course plots of the ACT scores, PEFR values and PM_2.5_, lagged by 1 day due to biological plausibility, are shown in figures1  2. While some participants appeared to have a close relationship between ACT and PM_2.5_ (eg, id-10, 11 and 19), there was no obvious visual relationship in most cases, and PEFR did not seem to vary substantially. The data for the patients with COPD (Bronkotest scores vs PM_2.5_) showed no consistent relationship (online supplemental figure S1).

**Figure 2:**
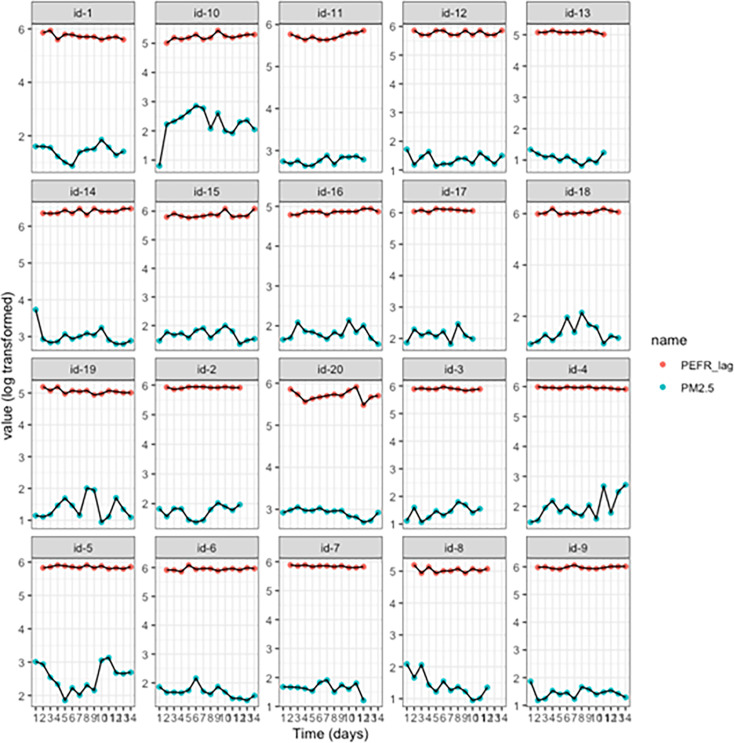
Time course monitoring of particulate matter (PM_2.5_) plotted against daily peak expiratory flow rate (PEFR) scores of participants. Each plot represents daily PEFR recorded and the daily average of PM_2.5_ measurements recorded for the study period. The values of PM_2.5_ and PEFR were log transformed to aid visualisation on one plot and to identify the time trends. (Refer to the online supplemental results for comparable figures and tables for actual values of PM_2.5_.)

### Impact of pollutants on asthma symptoms and PEFR

In assessing the relationship between symptom outcomes in asthma (PEFR values and ACT scores) and air quality, we chose to focus on PM_2.5_ because PM_10_ did not exhibit consistent patterns or peaks, and PM_2.5_ showed patterns similar to PM_1_. The lagged linear mixed-effects models and autoregressive models for ACT scores, PEFR values and PM_2.5_ are summarised in table 3 and demonstrate no significant association between PM_2.5_ and symptoms (p>0.05). Similar results were observed with unlagged models (see online supplemental table S1). The lagged models had a lower Akaike information criterion compared with the non-lagged models, indicating a relatively better fit. The low number of patients with COPD (Bronkotest scores vs PM_2.5_) precluded modelling.

**Table 3:** The association between PM_2.5_ and the symptoms (ACT/PEFR) in 20 patients with asthma

Models	Parameters	PM_2.5_ (linear mixed-effects models)	PM_2.5_ (autoregressive process models)
ACT	Coefficient (β)	0.083	0.100
	SE	0.058	0.059
	CI	(−0.03 to 0.20)	(−0.02 to 0.22)
	P value	0.158	0.092
PEFR	Coefficient (β)	−0.43	−0.477
	SE	0.712	0.700
	CI	(−1.83 to 0.98)	(−1.85 to 0.90)
	P value	0.549	0.496

Mixed linear models with lagged (by 1 day) symptoms and an autoregressive correlation structure of order 1 were applied. The models include random intercept and slope for time (day): ACT/PEFR (lagged) ~ PM_2.5_ + (1+ day | individual).

ACT, Asthma Control Test; PEFR, peak expiratory flow rate; PM, particulate matter.

## Discussion

Our study demonstrated that home monitoring of PM indoors was feasible in respiratory patients and influenced by smoking, such that future studies should control for this by design (eg, recruit non-smoking households). High levels of indoor air pollution were observed in many patients and appeared to relate to human activities, given the apparent link to normal mealtimes. This suggests that influencing indoor air is likely to be feasible through behavioural modification. However, we were unable to reproduce associations with clinical outcomes, likely due to the small sample size.

### Smoke exposure and indoor particulate levels

In COPD, current smoking made a significant difference to the level and pattern of particulate exposure—current smokers typically had levels significantly higher than the standardised thresholds set by the WHO; exposures to such high values were ‘normal’ per their daily routine.^18^ High concentrations of pollutants from smoking have been observed in other studies, although focused on partially enclosed outdoor hospitality venues where PM_2.5_ concentrations ranged from 10 µg/m^3^ to 90 µg/m^3^.^19 20^ In a review by Tong *et al* on the impact of secondhand smoke on air quality, most of the studies included focused on PM_2.5_ as a marker for secondhand smoke.^20^ However, this is a risky approach to take given that there are many other sources of particulates. For example, dissecting the increased risk of hospitalisation in patients with COPD exposed to high levels of PM_2.5_ would not be distinguishable from the known deleterious effects of current smoking.

### Other potential sources of PM

PM_2.5_ and PM_1_ peaks were often observed between 6 pm and 8 pm and 10 am and 12 noon. This is similar to the diurnal trends observed in the UK for PM_2.5_ both outdoors and indoors, with the highest mean concentrations occurring at 9 pm for urban background and urban traffic sites.^21 22^ While high PM_2.5_ emissions in the evening outdoors are attributed to domestic combustion from wood and coal for heating, the source of high peaks for our study participants is likely to be cooking since indoor solid fuel use was not reported by participants.^22^ The results are consistent with those of other studies, such as the one by Bousiotis *et al* which monitored the indoor air quality of a model family home in Worcestershire by placing low-cost sensors in different rooms (office, kitchen and bedroom) and one outdoors for 50 days.^23^ The study found that, although relatively lower concentrations were observed in the kitchen compared with other rooms, it had the highest PM concentrations during cooking periods.

Similarly, a study conducted in an occupied residence in Sweden found that concentrations of PM_1_ indoors were higher than outdoors, and that the principal sources of indoor particle composition were cooking and vaping.^24^ Factors such as the tools and ingredients used for cooking, cooking methods (worse with oil based (frying) vs water based (boiling)), energy sources (solid fuel>gas>electric), temperature (higher being worse) and surface area play a part.^25 26^ Wang *et al* also explored some specific ingredients that drove emission levels.^27^ The study revealed that particles discharged from non-meat ingredients (eg, cabbage, potatoes) were higher in number than those from meats. Additives such as table salt and black pepper also appeared to decrease PM_2.5_ emission rates in cooking oils, especially when added at the beginning of cooking.^25^ The HOMEChem study by Patel *et al* probed the indoor chemistry in a manufactured test house and found that exposure to PM_2.5_ concentrations was higher after cooking than during cooking and could rise to 250 μg/m^3^, irrespective of the meal being prepared, suggesting that peaks observed in our data represent emissions during and after meal preparation.^28^ Although cooking may be an important activity exposing most participants to high levels of PM_2.5_, specific details about what constitutes cooking cannot be inferred from our findings.

### Diurnal patterns of pollutant exposure

Low levels of PM_2.5_ outdoor emissions have been observed between 12 am and 6 am at urban background and roadside sites, consistent with the low levels observed overnight in our data.^22^ Fluctuations in our study (particularly during other periods of the day) appear to be primarily triggered by human activities, due to timing and because emissions from buildings and furnishings are relatively stable.^19^ Consistent with mainly indoor sources from human activity, in the study by Bousiotis *et al*, the pollutants in the home were assessed during a period of the family’s absence.^23^ PM concentrations were uniform and subject to only outdoor sources in their absence, but indoor sources could be located when they were present. Bousiotis *et al* further reported observing peaks in pollutant levels early in the morning (at 06:00) and late at night (at 23:00), particularly in the bedroom, in their study. These peaks were associated with family members waking up and adults preparing for bedtime, respectively.

### Impact of PM_2.5_ exposure on asthma outcomes

Our results demonstrate significant exposure to indoor PM, likely derived mainly from human activities that may adversely influence markers of asthma control in some patients. This, however, did not seem to relate to clinical outcomes—the small cohort size and limited number of measures available hindered our ability to prove this statistically, and to include other characteristics that could influence the ACT scores and the PEFR values (such as smoking status, age, etc) in modelling. Similar findings can be seen in a systematic review by Paterson *et al* of studies assessing the impact of indoor PM_2.5_ and VOC exposure on asthma symptoms, where PM_2.5_ did not relate to symptoms, unlike VOCs.^29^ The significant heterogeneity between the reviewed studies could have informed the study findings by Paterson *et al.* Additionally, most of these studies were cross-sectional.

It is also worth noting that though most participants in our study were at the severe stages of asthma and COPD, several of them were on multiple anti-inflammatory medications such as montelukast, inhaled corticosteroids and biologics. Trenga *et al* found that exposure to PM_2.5_ was associated with a decrease in PEFR in children with asthma who were not taking anti-inflammatory medications.^30^ This concept may explain the less pronounced effect observed in our patients. In addition, variability in participants’ responses to pollutants could be influenced by the phenotypes and disease severity of participants.^31^ Anti-inflammatory medications may dampen airway response to PM_2.5_, hence differing from less severe disease with lower medication burden. Another demographic factor that may impact overall exposure levels (although not those measured in the home in our study) is employment; this was less likely to be a confounder in our cohort where only 33% were actively employed.

### Strengths and limitations

Strengths include participant recruitment from a wide geographical area, including areas of deprivation. The recruitment numbers are representative of the prevalence of asthma and COPD in England, with patients with asthma outnumbering those with COPD.^32 33^ PM measures in the study were reliable, with a high correlation with the reference device.^34^ However, the failure of the Flow 2 device with respect to NO_2_ and VOC measures was a significant limitation, because the combined effect of multipollutant exposure levels on lung function is essential.^35^ Studies such as Lee *et al* demonstrated that increased levels of multiple pollutant exposure potentially lead to the development of small airway diseases, which increases the risk of exacerbations of airway disease.^35^ We were unable to accurately assess this since the monitor and supersite data failed to align. The most serious limitation was small sample size, although this was expected as an exploratory preliminary work.

In addition, ACT has not been validated as a tool for daily responsiveness, with validation studies using a 4-week recall period.^36^ However, the nature of the questions lends itself to daily use, and participants were informed to report in this manner. Bronkotest has been used successfully in daily monitoring by our group and in trials, although for the purpose of acute exacerbation of COPD (AECOPD) recognition.^37 38^ Participants also generally failed to report activities taken to alter indoor exposure, such that these could not be accounted for, and we did not include indoor-outdoor air quality modelling, because of the small nature of our study and uncertainty on the method by which indoor-outdoor interfaces should be modelled statistically. For instance, this might vary with house construction and window opening.

## Conclusion

In conclusion, our data confirm that human activities likely drive indoor air quality for PM, suggesting that behavioural interventions may optimise this. While we were unable to show a relationship between disease outcomes and indoor air quality, this may relate to sample size, given that many other studies support an adverse relationship existing. Two ongoing projects in the UK, using mixed methods and codesign (INGENIOUS and SAMHE), are likely to provide valuable insights into optimising indoor air quality through behavioural change.^39 40^ Larger studies with direct measurement indoors, in non-smokers, focusing on asthma rather than a combined airway disease cohort, and perhaps in less severe populations with fewer medications will be required to determine if symptom variation is directly linked to exposure. Understanding which components of PM are driving any symptom association will also be vital to ensure that behaviour change is targeted to areas of maximum putative benefit. For example, high proportions of bioaerosols, along with individual sensitisation to this, would prioritise measures targeting atopic disease and damp housing.

## Supplementary material

10.1136/bmjresp-2025-003807 online supplemental file 1

## Data Availability

Data are available upon reasonable request. All data relevant to the study are included in the article or uploaded as supplementary information.
