# Supplementary material for: Indoor air quality and its impacts on asthma and COPD
Source: BMJ Open Respir Res. 2026 Mar 18;13(1):e003807. doi: 10.1136/bmjresp-2025-003807 (PMC13007101; doi:10.1136/bmjresp-2025-003807)
Supplement: online supplemental file 1 [file bmjresp-13-1-s001.docx]

**SUPPLEMENTARY RESULTS**


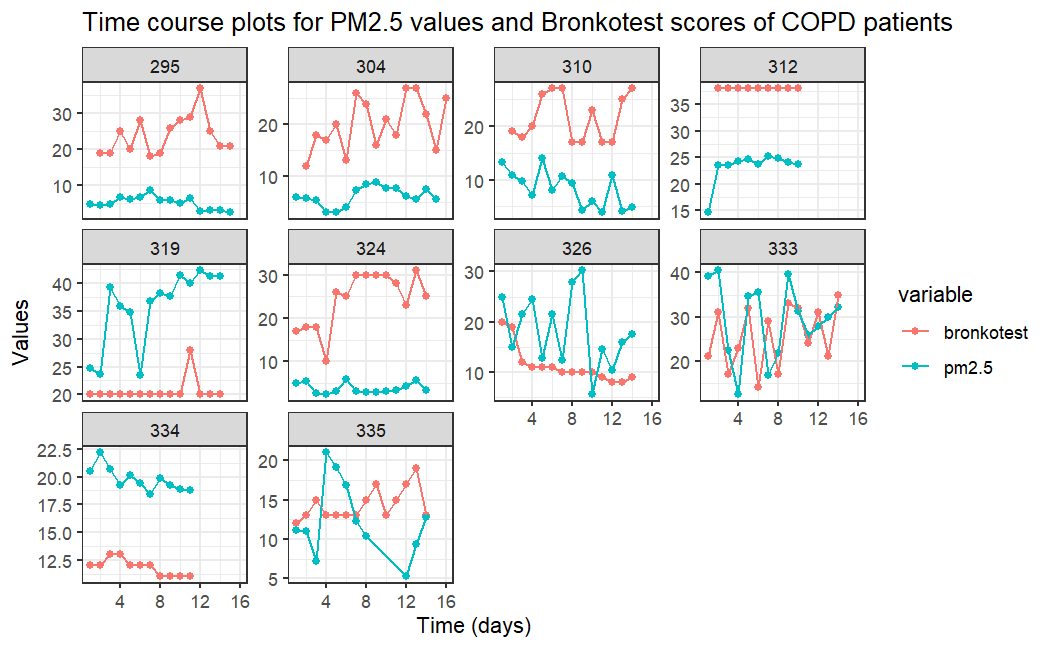


***Figure S1****: Bronkotest time course plots of daily average of PM_2.5_ values and Bronkotest scores recorded by COPD participants within study period. Each plot represents daily average of the PM_2.5_ values recorded by the air quality monitor and the daily Bronkotest score recorded for the study period for respective COPD participants.*


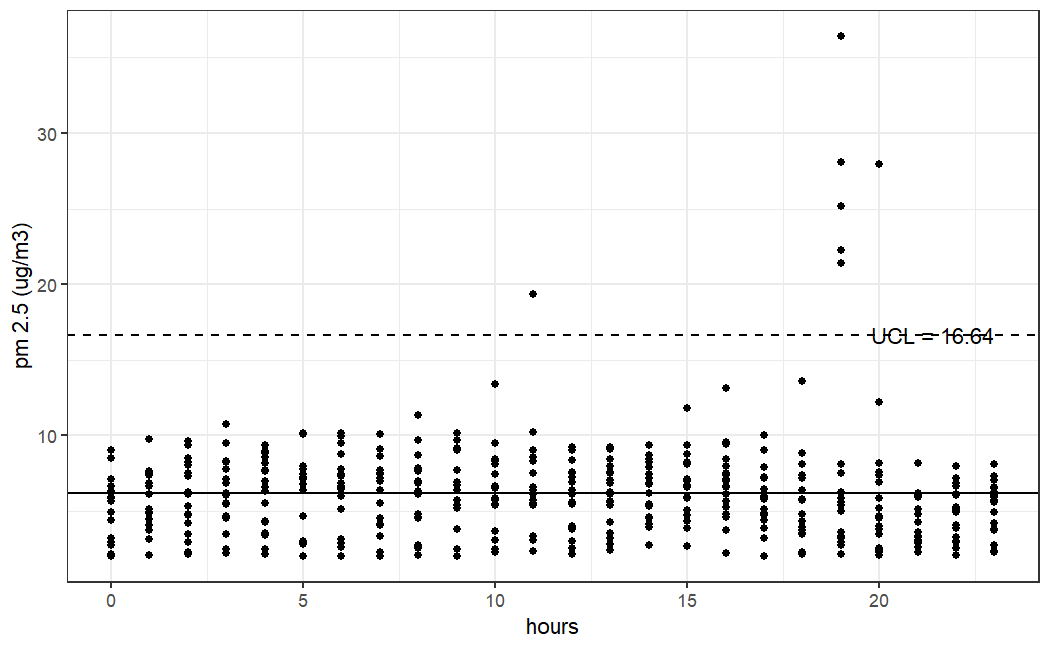


***Figure S2****: Representative statistical process control chart of the PM_2.5_ data of a non-smoker. Each plot represents an hourly average of minute-by-minute measurements of every pollutant for at least 14 days. The line between several plots represents the mean while the dashed line above most plots represents the upper control limit (UCL) of each pollutant measured. No lower control limit appears on the representative charts because these were negative in most cases, which is not possible, and simply reflects the wide variance above 0.*


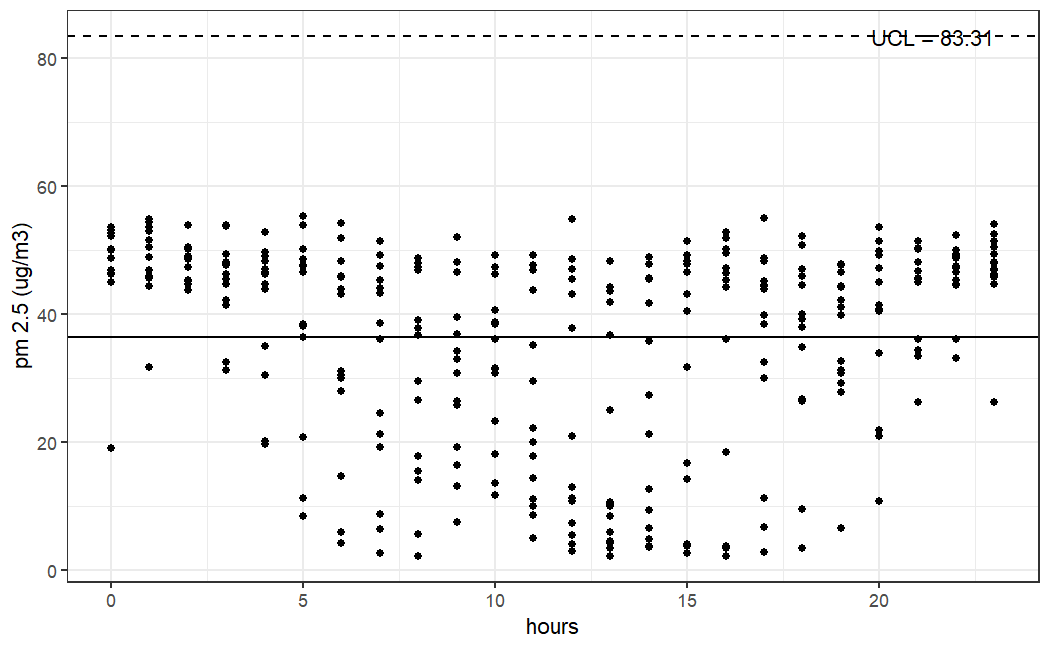


***Figure S3****: Representative statistical process control chart of the PM_2.5_ data of a smoker. Each plot represents an hourly average of minute-by-minute measurements of every pollutant for at least 14 days. The line between several plots represents the mean while the dashed line above most plots represents the upper control limit (UCL) of each pollutant measured. No lower control limit appears on the representative charts because these were negative in most cases, which is not possible, and simply reflects the wide variance above 0.*


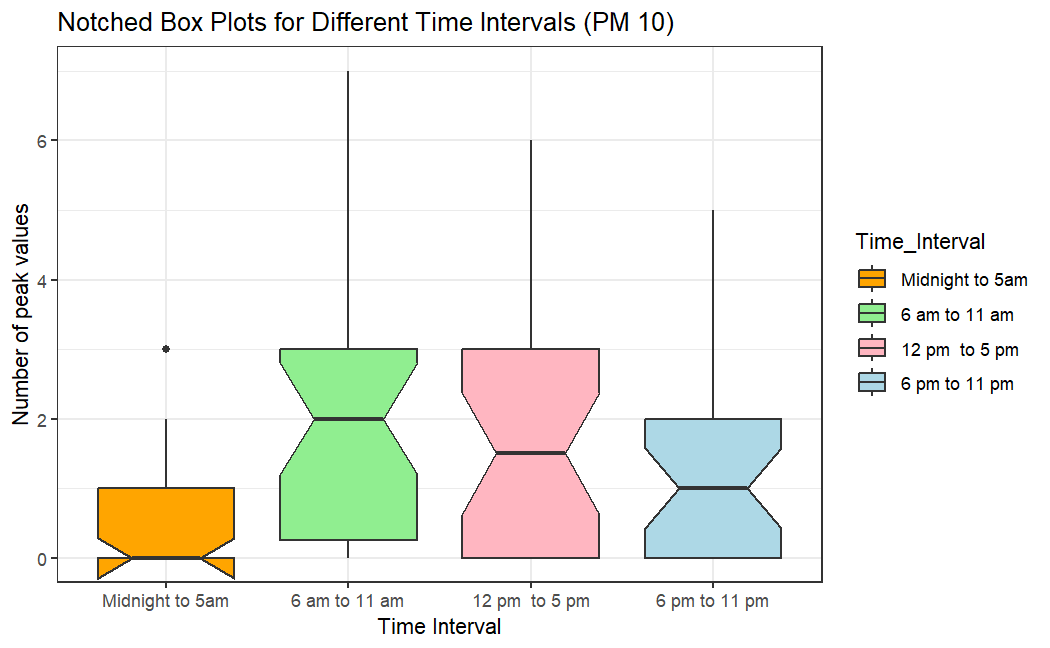


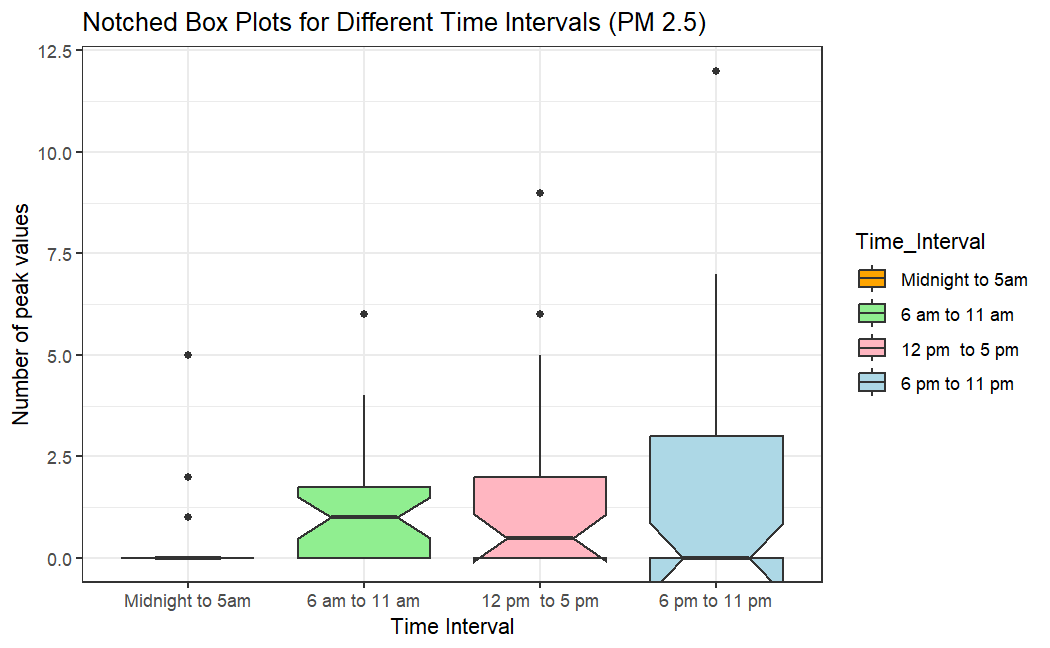


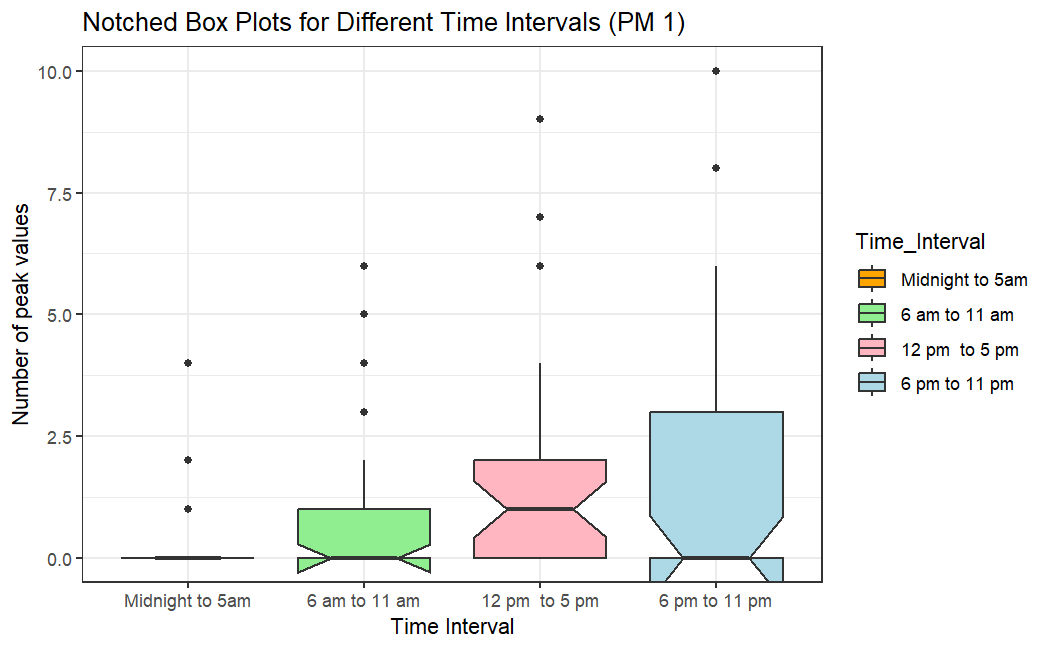


***Figure S4****: Notched box plots with 95% confidence interval for PM_10_, PM_2.5_ and PM_1._ Significant differences between the time intervals are observed in the notches of the box plots not overlapping.*

**Association between PM_2.5_ exposure and symptoms**

| Models | Parameters | PM_2.5_ (linear mixed effect models) | PM_2.5_ (Autoregressive process models) |
| --- | --- | --- | --- |
| ACT | Coefficient (β) | -0.035 | -0.044 |
|  | Standard Error | 0.046 | 0.046 |
|  | Confidence Interval | (-0.13, 0.06) | (-0.13, 0.05) |
|  | P- value | 0.441 | 0.334 |
| PEFR | Coefficient (β) | -0.60 | -0.600 |
|  | Standard Error | 0.55 | 0.550 |
|  | Confidence Interval | (-1.68, 0.48 ) | (-1.68, 0.48) |
|  | P- value | 0.276 | 0.277 |

***Table S1****: Summary of the association between PM_2.5_ and the symptoms (ACT/PEFR) in 20 asthma patients. Mixed linear models with symptoms and an autoregressive correlation structure of order 1 were applied.*

**Peak counts at 6-hourly time intervals and upper limit values for all participants**

| Patient ID | Pollutants | Time Intervals  (Hours) | | | | | | | Total peaks > UCL | UCL  Values  (µg/m^3^) |
| --- | --- | --- | --- | --- | --- | --- | --- | --- | --- | --- |
|  |  | 0-5 | 6-11 | | 12-17 | 18-23 | | |  |  |
| 267 | PM_10_ | - | 3 | 2 | | | 3 | | 8 | 66.4 |
|  | PM_2.5_ | - | 1 | - | | | 6 | | 7 | 11 |
|  | PM_1_ | - | - | - | | | 6 | | 6 | 5.5 |
| 270 | PM_10_ | 1 | 7 | - | | | 2 | | 10 | 59.31 |
|  | PM_2.5_ | - | 2 | 1 | | | 1 | | 4 | 14 |
|  | PM_1_ | - | 2 | 1 | | | 1 | | 4 | 7.6 |
| 280 | PM_10_ | - | 3 | 4 | | | - | | 7 | 58 |
|  | PM_2.5_ | - | 1 | 4 | | | - | | 5 | 9.7 |
|  | PM_1_ | 1 | - | 2 | | | - | | 3 | 3.2 |
| 288 | PM_10_ | - | - | | 4 | 3 | | 7 | | 79.5 |
|  | PM_2.5_ | - | - | | - | 2 | | 2 | | 14.4 |
|  | PM_1_ | - | - | | 1 | 3 | | 4 | | 5.5 |
| 292 | PM_10_ | 1 | 3 | | 2 | - | | 6 | | 84.2 |
|  | PM_2.5_ | - | 4 | | 6 | - | | 10 | | 35.6 |
|  | PM_1_ | - | 4 | | 6 | - | | 10 | | 26.4 |
| 293 | PM_10_ | - | - | | 3 | - | | 3 | | 109.3 |
|  | PM_2.5_ | - | - | | 6 | - | | 6 | | 51 |
|  | PM_1_ | - | 1 | | 7 | 2 | | 10 | | 36.2 |
| 294 | PM_10_ | 2 | 7 | - | | | - | | 9 | 77.6 |
|  | PM_2.5_ | 2 | 6 | - | | | - | | 8 | 9.9 |
|  | PM_1_ | 1 | 6 | - | | | - | | 7 | 2.7 |
| 295 | PM_10_ | - | 3 | 2 | | | 2 | | 7 | 53.8 |
|  | PM_2.5_ | - | - | - | | | 2 | | 2 | 12.6 |
|  | PM_1_ | - | - | 1 | | | 1 | | 2 | 5.1 |
| 297 | PM_10_ | 1 | 7 | 1 | | | 1 | | 10 | 56 |
|  | PM_2.5_ | - | 4 | 1 | | | 1 | | 6 | 17.7 |
|  | PM_1_ | - | 5 | 1 | | | - | | 6 | 11.7 |
| 303 | PM_10_ | - | 1 | 2 | | | 5 | | 8 | 63.2 |
|  | PM_2.5_ | - | - | - | | | 3 | | 3 | 12.2 |
|  | PM_1_ | - | - | - | | | 3 | | 3 | 6.4 |
| 304 | PM_10_ | 1 | 3 | - | | | 3 | | 7 | 74.1 |
|  | PM_2.5_ | - | 1 | - | | | 6 | | 7 | 16.6 |
|  | PM_1_ | - | 1 | - | | | 6 | | 7 | 8 |
| 305 | PM_10_ | 3 | 2 | - | | | - | | 5 | 183 |
|  | PM_2.5_ | 1 | - | - | | | - | | 1 | 38.1 |
|  | PM_1_ | 2 | - | - | | | - | | 2 | 21.8 |
| 306 | PM_10_ | 1 | 7 | 3 | | | - | | 11 | 127.4 |
|  | PM_2.5_ | 5 | 4 | 1 | | | - | | 10 | 33.2 |
|  | PM_1_ | 4 | 4 | 1 | | | - | | 9 | 20.6 |
| 307 | PM_10_ | - | - | 5 | | | 2 | | 7 | 43.2 |
|  | PM_2.5_ | - | 1 | 2 | | | 4 | | 7 | 9.7 |
|  | PM_1_ | - | 1 | 2 | | | 3 | | 6 | 4.7 |
| 308 | PM_10_ | - | 3 | 3 | | | - | | 6 | 42.7 |
|  | PM_2.5_ | - | 3 | 1 | | | - | | 4 | 7.2 |
|  | PM_1_ | - | 4 | - | | | - | | 4 | 2.9 |
| 310 | PM_10_ | - | - | 1 | | | 2 | | 3 | 105.5 |
|  | PM_2.5_ | - | - | 1 | | | 5 | | 6 | 38.3 |
|  | PM_1_ | - | - | 1 | | | 8 | | 9 | 24.3 |
| 311 | PM_10_ | - | 1 | 1 | | | 3 | | 5 | 160.7 |
|  | PM_2.5_ | - | - | - | | | 7 | | 7 | 43.5 |
|  | PM_1_ | - | - | - | | | 6 | | 6 | 28.1 |
| 312 | PM_10_ | 2 | - | 1 | | | - | | 3 | 98.6 |
|  | PM_2.5_ | - | - | - | | | - | | 0 | 32.9 |
|  | PM_1_ | - | - | - | | | - | | 0 | 17.3 |
| 313 | PM_10_ | 1 | 5 | 3 | | | - | | 9 | 70.7 |
|  | PM_2.5_ | - | - | 2 | | | - | | 2 | 10.7 |
|  | PM_1_ | - | - | 2 | | | - | | 2 | 3.6 |
| 314 | PM_10_ | - | 2 | 2 | | | 1 | | 5 | 84.6 |
|  | PM_2.5_ | - | - | 9 | | | - | | 9 | 19.5 |
|  | PM_1_ | - | - | 9 | | | - | | 9 | 11.2 |
| 315 | PM_10_ | - | 2 | 3 | | | - | | 5 | 73.3 |
|  | PM_2.5_ | - | 1 | 5 | | | 3 | | 9 | 26.4 |
|  | PM_1_ | - | 1 | 6 | | | 3 | | 10 | 20.9 |
| 318 | PM_10_ | 1 | 1 | 4 | | | 1 | | 7 | 71.2 |
|  | PM_2.5_ | - | 1 | 2 | | | 4 | | 7 | 16.5 |
|  | PM_1_ | - | - | 2 | | | 5 | | 7 | 11.2 |
| 319* | PM_10_ | - | - | - | | | 3 | | 3 | 191 |
|  | PM_2.5_ | - | - | - | | | - | | 0 | 83.3 |
|  | PM_1_ | - | - | - | | | - | | 0 | 56.5 |
| 323 | PM_10_ | 2 | - | - | | | 4 | | 6 | 64.2 |
|  | PM_2.5_ | 1 | - | - | | | 12 | | 13 | 16.5 |
|  | PM_1_ | 1 | - | - | | | 10 | | 11 | 9.3 |
| 324 | PM_10_ | - | 1 | 6 | | | 1 | | 8 | 62.9 |
|  | PM_2.5_ | - | 3 | 4 | | | - | | 7 | 15.1 |
|  | PM_1_ | - | 3 | 4 | | | - | | 7 | 9.8 |
| 326* | PM_10_ | - | 2 | 2 | | | 2 | | 6 | 143.8 |
|  | PM_2.5_ | - | - | - | | | - | | 0 | 66.4 |
|  | PM_1_ | - | - | - | | | - | | 0 | 47.2 |
| 328 | PM_10_ | 2 | 6 | - | | | - | | 8 | 119.7 |
|  | PM_2.5_ | - | - | - | | | - | | 0 | 25.7 |
|  | PM_1_ | - | - | - | | | - | | 0 | 14.3 |
| 333* | PM_10_ | - | - | - | | | 1 | | 1 | 280.9 |
|  | PM_2.5_ | - | 1 | - | | | - | | 1 | 78.1 |
|  | PM_1_ | - | 1 | - | | | - | | 1 | 55 |
| 334 | PM_10_ | - | 2 | 1 | | | - | | 3 | 148.4 |
|  | PM_2.5_ | - | 1 | 4 | | | - | | 5 | 30 |
|  | PM_1_ | - | 1 | 3 | | | - | | 4 | 16.7 |
| 335 | PM_10_ | - | 2 | - | | | - | | 2 | 57.1 |
|  | PM_2.5_ | - | 2 | - | | | - | | 2 | 51.1 |
|  | PM_1_ | - | - | - | | | - | | 0 | 41.4 |

***Table S2****: Summary table of all counts of individual peaks above the upper limits for all pollutants recorded at specific time intervals for all participants. UCL- upper control limit. *Participants among this cohort who were smokers.*

**ACT (Asthma Control Test)**

| Questions | Score |
| --- | --- |
| During past 4 weeks, how much of the time has your asthma kept you from getting things done at work, home or school? | 1.All the time  2.Most of the time  3.Some of the time  4.A little of the time  5.None of the time |
| During past 4 weeks, how often have you had shortness of breath? | 1.More than once a day  2.Once a day  3.3-6 times a week  4.1-2 times a week  5.None |
| During past 4 weeks, how often have your asthma symptoms woken you up in the night or earlier than usual in the morning? | 1.4 or more times a week  2.2 or 3 night a week  3.Once a week  4. Once or twice  Not at all |
| During past 4 weeks, how often have you used your rescue inhaler or nebuliser medication (such as  Salbutamol)? | 1.3 or more times a day  2.1-2 times a day  3.2-3 times a week  4.Once a week or less  5.Not at all |
| During past 4 weeks, how would you rate your asthma control during the last 24 hours? | 1.Not controlled at all  2.Poorly Controlled  3.Somewhat Controlled  4.Well Controlled  5.Completely Controlled |

***Table S3****: Asthma control test assessing disease control and symptoms of participants with asthma*

**Bronkotest Questionnaire**

| Questions | Score |
| --- | --- |
| Describe your breathing | 1.Better than usual  2.Normal/Usual  3.Worse than usual  4.Much worse than usual |
| The amount of sputum you produce | 0.None  1.Some  2.A little  3.Moderate  4.A lot |
| Type of sputum | 1.Watery  2.Sticky liquid  3.Semi-solid  4.Solid |
| How do you feel? | 1.Better than usual  2.Normal/usual  3.Worse than usual  4.Much worse than usual |
| How often do you cough? | 0.Rarely  1.Occassionally  2.Frequently  3.Persistently |

***Table S4****: Bronkotest questionnaire assessing disease control and symptoms of participants with COPD*

**Peak values above UCLs compared with WHO Air Quality Guidelines**

| Pollutants | Mean of peak counts above upper limits | Mean of upper limits  (µg/m^3^) | WHO Annual Average (µg/m^3^) | WHO 24-hour Average (µg/m^3^) |
| --- | --- | --- | --- | --- |
| PM_10_ | 6.17 (±2.61) | 97.01 (±53.27) | 15 | 45 |
| PM_2.5_ | 5 (±3.5) | 28.23 (±20.59) | 5 | 15 |
| PM_1_ | 4.97 (±3.52) | 17.83 (±15.54) | - | - |

***Table S5****: Average mean values of upper limits and the average number of peaks above them counted compared with WHO air quality guidelines*
